# Genome-Wide Identification and Expression Analysis of the GRAS Gene Family and Their Responses to Heat Stress in *Cymbidium goeringii*

**DOI:** 10.3390/ijms25126363

**Published:** 2024-06-08

**Authors:** Ye Huang, Qinyao Zheng, Meng-Meng Zhang, Xin He, Xuewei Zhao, Linying Wang, Siren Lan, Zhong-Jian Liu

**Affiliations:** 1 Key Laboratory of National Forestry and Grassland Administration for Orchid Conservation and Utilization at College of Landscape Architecture, Fujian Agriculture and Forestry University, Fuzhou 350002, China; yehuang0809@fafu.edu.cn (Y.H.); qinyaozheng@fafu.edu.cn (Q.Z.); linyingwang@fafu.edu.cn (L.W.); 2 College of Forestry, Fujian Agriculture and Forestry University, Fuzhou 350002, China; 1220428020@fafu.edu.cn (M.-M.Z.); 5220422102@fafu.edu.cn (X.H.); zxw6681@163.com (X.Z.)

**Keywords:** GRAS gene family, *Cymbidum goeringii*, heat stress, expression analysis

## Abstract

The GRAS gene family, responsible for encoding transcription factors, serves pivotal functions in plant development, growth, and responses to stress. The exploration of the GRAS gene family within the Orchidaceae has been comparatively limited, despite its identification and functional description in various plant species. This study aimed to conduct a thorough examination of the GRAS gene family in *Cymbidum goeringii*, focusing on its physicochemical attributes, phylogenetic associations, gene structure, cis-acting elements, and expression profiles under heat stress. The results show that a total of 54 *CgGRAS*s were pinpointed from the genome repository and categorized into ten subfamilies via phylogenetic associations. Assessment of gene sequence and structure disclosed the prevalent existence of the VHIID domain in most *CgGRAS*s, with around 57.41% (31/54) *CgGRAS*s lacking introns. The Ka/Ks ratios of all *CgGRAS*s were below one, indicating purifying selection across all *CgGRASs*. Examination of cis-acting elements unveiled the presence of numerous elements linked to light response, plant hormone signaling, and stress responsiveness. Furthermore, *CgGRAS5* contained the highest quantity of cis-acting elements linked to stress response. Experimental results from RT-qPCR demonstrated notable variations in the expression levels of eight *CgGRAS*s after heat stress conditions, particularly within the LAS, HAM, and SCL4/7 subfamilies. In conclusion, this study revealed the expression pattern of *CgGRAS*s under heat stress, providing reference for further exploration into the roles of *CgGRAS* transcription factors in stress adaptation.

## 1. Introduction

In recent years, an increasing volume of research has shown that GAI-RGA- and -SCR (GRAS) transcription factors have been recognized for their versatile functions in plant growth, development, and defense mechanisms against diverse biotic and abiotic stressors [1]. The acronym GRAS stems from the initial disco very of three proteins: GIBBERELLIN-ACID INSENSITIVE (GAI), REPRESSOR of GA1 (RGA), and SCARECROW (SCR) [2]. These transcription factors are characterized by a conserved GRAS domain at the C-terminus, featuring motifs such as LHR I (Leucine Heptad Repeat Ⅰ), LHR Ⅱ (Leucine Heptad Repeat Ⅱ), VHIID, PFYRE, and SAW. These transcription factors also have a variable N-terminus that includes amino acid homopolymers, intrinsically disordered proteins (IDD), and the universally conserved DELLA domain [3,4]. Typically, GRAS proteins range from 400 to 700 amino acids in length and share a high degree of sequence similarity in their C-terminal region [5].

So far, homologs of GRAS genes have been discovered in numerous higher plants, attracting extensive research interest regarding their functions. Previously, the GRAS genes have been identified across a variety of plant species, including *Arabidopsis thaliana* [6], *Oryza sativa* [7], Chinese cabbage [8], soybean [9], *Prunus mume* [10], *Nelumbo nucifera* [11], etc. Numerous studies have identified varying numbers of subfamilies within the GRAS gene family [9,12,13,14]. Based on variations in N-terminal length and sequence, GRAS gene family members can be categorized into a maximum of 17 subfamilies (LISCL, PAT1, SHR, HAM, SCR, LAS, SCL4/7, SCL3, DELLA, NSP1, NSP2, SCLA, SCLB, RAD1, SCL32, RAM1, and DL) [15].

Firstly, GRAS genes are implicated in a range of essential plant growth and developmental activities. For instance, the LAS subfamily inhibits the formation of axillary meristems, directing growth toward the meristems [16]. Homoeologous GRAS transcription factors, AhNSP2-B07 (Nb) and AhNSP2-A08 (Na), were shown to regulate nodulation in cultivated peanuts [17]. Additionally, an SCR/SHR complex, comprising the SCR and SHR, participates in the radial organization of roots and stems [18,19]. Within the realm of plant hormone signal transduction, three Arabidopsis GRAS genes (*AtPAT1*, *AtSCL5*, and *AtSCL21*) act as positive regulators of phytochrome-A, while *AtSCL13* plays a key role in phytochrome-B signal transduction in *A. thaliana* [20,21,22]. The SCL3 subfamily and the DELLA subfamily counteract each other, modulating GA (gibberellin) signal transduction in plants, thus controlling processes such as seed, stem, and flower development [23]. Significantly, GRAS proteins are also integral components in both biotic and abiotic stress responses. *GmGRAS37* boosted drought and salt tolerance in transgenic plants by inducing the expression of *GmDREB1*, *GmNCED3*, *GmCLC1*, *GmSOS1*, *GmSOD1*, and *GmSOD2* [9]. HAM and the PAT1 subfamily have been linked to responses to drought and salt stress in *Fragaria vesca*, engaging through various mechanisms to react to different abiotic stresses [24]. The ectopic expression of *VaPAT1* in *A. thaliana* altered the expressions of a range of stress-related genes, enhancing tolerance to cold, drought, and high salinity in VaPAT1-overexpression lines [25]. Although the GRAS gene family has been studied for many years, we currently have only a limited understanding of many aspects, such as the expansion mechanism and stress breeding of this gene family in flowering plants. In addition, most of these studies on the GRAS gene family focus on the response to cold, drought, and salt stress, and there is a lack of exploration of heat stress. In contrast, studies on the evolutionary relationships and functional characteristics of the GRAS gene family in Orchidaceae are scarce.

The Orchidaceae exhibit remarkable variation in aspects such as the regulation of flowering, color presentation, scent production, and mycorrhizal relationships [26,27,28,29]. While extensive research has probed the GRAS gene family in model plants, the characteristics and roles of GRAS genes within the Orchidaceae remain largely unexplored. In recent years, due to habitat degradation and excessive collection by plant sellers and enthusiasts, *Cymbidium. goeringii* has been listed as an endangered species, so it is necessary to study the response mechanism of *C. goeringii* to abiotic stress. The research conducted by Zeng et al. led to the identification of 47 GRAS genes in *Dendrobium. catenatum*, with a specific emphasis on the LISCL, HAM, and LAS subfamilies, which were found to be highly reactive to abiotic stresses like drought and cold [5]. Furthermore, in *Dendrobium. chrysotoxum*, sensitivity to stress was observed in members of the LISCL, LAS, and DELLA subfamilies [30]. The advent of sequencing technologies has enabled the reporting of several orchid genomes, offering a foundation for analyzing orchid GRAS gene families. A thorough examination of the GRAS gene family in the complete genomes of *C. goeringii* holds significant implications for the development and utilization of this orchid species.

This study systematically identified 54 GRAS gene members in *C. goeringii*. We analyzed their gene structures, constructed phylogenetic trees, conducted cis-acting element analyses, and specifically analyzed their expression patterns under heat stress. The above studies were used to enrich the research gap in the GRAS gene family on heat stress in orchids. Our findings contribute to expanding our comprehension of the evolutionary relationship and functional attributes of the GRAS family in orchids. This study also provides some new insights for further investigating the roles of *CgGRAS* proteins and stress breeding.

## 2. Results

### 2.1. Identification and Physicochemical Properties of the GRAS Gene Family in C. goeringii

A total of 54 GRAS genes were identified by using the local BLAST program and the Pfam tool to search the whole genome of *C. goeringii*, named *CgGRAS1*–*CgGRAS54* based on their distribution order of genes on chromosomes. In addition, we also used the Expasy online tool to analyze the physicochemical properties of CgGRASs (Table S1). The derived GRAS proteins varied greatly in amino acid number (AA), with lengths ranging from 73aa (CgGRAS14) to 849aa (CgGRAS9), averaging 424aa. The molecular weight (MW) of the 54 GRAS genes ranged from 5.51 kDa (CgGRAS29) to 97.33 kDa (CgGRAS9), with an average value of 46.94 kDa. The isoelectric point (pI) range was 4.63 (CgGRAS29) to 11.18 (CgGRAS32), and the isoelectric point of 44 CgGRASs was less than 7.0, which was acidic. The remaining ten CgGRASs all have isoelectric points greater than 7.0 and are alkaline. The calculated grand average of hydrophilic values (GRAVY) ranges from −0.647 (CgGRAS41) to 0.166 (CgGRAS23), mostly indicating hydrophilic properties (GRAVY < 0). The instability index (II) varied from 25.79 (CgGRAS29) to 88.71 (CgGRAS32), while the aliphatic index (AI) ranged from 66.4 (CgGRAS29) to 106.38 (CgGRAS52).

### 2.2. Phylogenetic Analysis of GRAS Genes

**Figure 1:**
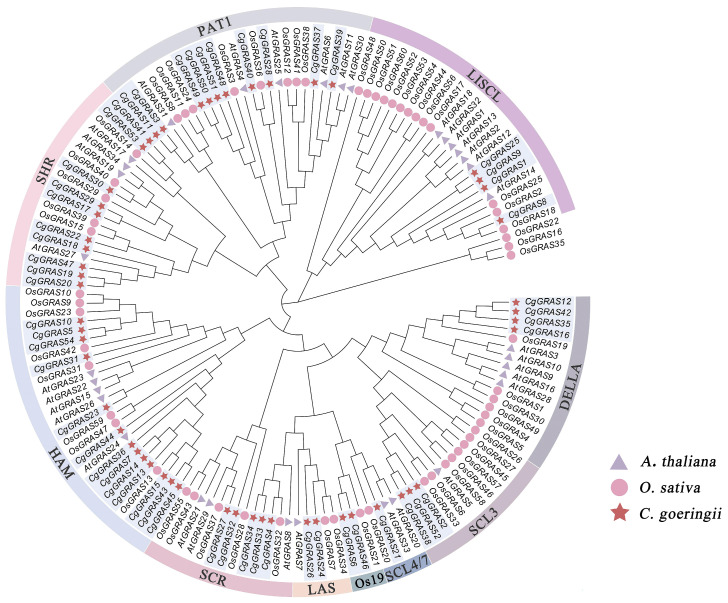
Phylogenetic tree of GRAS genes based on the GRAS protein sequences of *A. thaliana*, *O. sativa*, and *C. goeringii*.

To analyze and classify the phylogenetic relationships of GRAS genes in *C. goeringii*, we constructed a phylogenetic tree using 54 GRAS genes from *C. goeringii*, along with 34 and 60 GRAS genes from *A. thaliana* and *O. sativa*, respectively, identified in this study. The 148 GRAS genes were divided into ten subfamilies: LISCL (4 genes), PAT1 (10 genes), SHR (10 genes), HAM (13 genes), SCR (5 genes), LAS (2 genes), Os19 (2 genes), SCL4/7 (1 gene), SCL3 (3 genes), and DELLA (4 genes) (Figure 1). Notably, the HAM subfamily had the highest membership, containing 13 GRAS genes; the SCL4/7 subfamily contains the smallest number of members, only one gene.

### 2.3. Gene Structure and Motif Analysis of GRAS Genes

**Figure 2:**
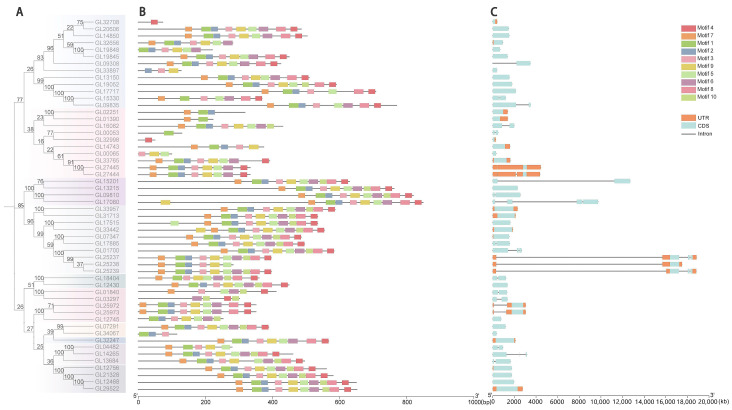
Analysis of the motif and gene structure of the GRAS gene family in *C. goeringii*. (**A**) Phylogenetic tree of 54 *CgGRAS*s. (**B**) Determination of conserved motifs in the *CgGRAS* proteins. (**C**) Gene structure of *CgGRAS*s.

For deeper insight into the genetic structure of the GRAS gene of *C. goeringii*, we utilized the MEME website to analyze its conserved protein motifs, denoted as motif1–motif10, and obtained their sequence and logo (Table S2). It was observed that the majority of conserved motifs within the GRAS gene were located in the C-terminal domain. Organized in the sequence of motif4, motif7, motif1, motif2, motif3, motif9, motif5, motif6, motif8, and motif10 (Figure 2B), among them, motif4, motif7, and motif1 are the most conserved. LISCL, Os19, and DELLA subfamilies have no motif deletion and a very stable conserved C-terminal domain, and each gene of the PAT1 subfamily also contains ten conserved motifs, except CgGRAS49. Only three CgGRASs showed motif increases (CgGRAS9, CgGRAS40, and CgGRAS28).

**Figure 3:**
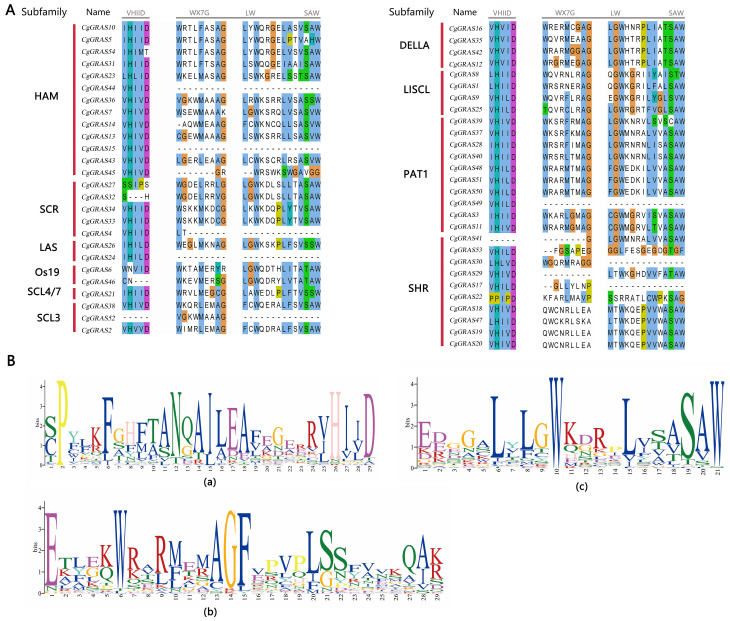
VHIID and SAW motifs in the GRAS protein amino acid sequences. (**A**) *CgGRAS* protein sequence alignment results. (**B**) Sequence logo of motifs. (a) Sequence logo of VHIID domain. (b) and (c): Sequence logo of SAW domain.

The intron–exon structure of the GRAS gene of *C. goeringii* was analyzed to further understand its characteristics (Figure 2C). The findings revealed that 57.41% of the genes lacked introns, and only 23 genes contained introns, of which three genes had long introns (*CgGRAS49*, *CgGRAS50*, and *CgGRAS51*), and all these genes belonged to the PAT1 subfamily. Among the 54 GRAS genes, intron numbers ranged from zero to three, while exon numbers ranged from one to four. *CgGRAS27* and *CgGRAS50* have similar amino acid lengths (410aa and 397aa), but a big difference is presented in gene lengths (1.8 kb and 19 kb). Furthermore, we conducted a sequence comparison of *CgGRAS* proteins, revealing the prevalence of the VHIID domain in most *CgGRAS* proteins, characterized by stable aspartic acid (D), with nearly all subfamilies exhibiting some degree of base substitution (Figure 3). Interestingly, all members of the DELLA family showed substitution of isoleucine (I) by valine (V), but all members have an extremely stable SAW domain.

### 2.4. Collinearity and Chromosomal Localization of CgGRAS Genes

**Figure 4:**
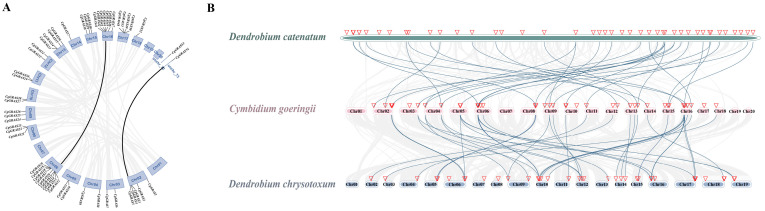
The collinearity analysis in C. goeringii, D. chrysotoxum, and D. catenatum. (**A**) Synteny analysis of C. goeringii. Black lines represent segmental duplicated gene pairs. (**B**) Collinearity analysis of GRAS genes of D. chrysotoxum, C. goeringii, and D. catenatum. The location of the SPLs is marked by a red triangle and the blue lines show GRAS genes with collinear relationships between different species.

Furthermore, an investigation into the collinearity among the GRAS gene sequences of *D. catenatum*, *C. goeringii*, and *D. chrysotoxum* was conducted, aimed at discerning potential gene duplication events pivotal in the evolutionary trajectory of the GRAS gene family within orchids (Figure 4). Comparative genomic data analysis identified 44 collinear gene pairs between *D. chrysotoxum* and *C. goeringii*, as well as 36 collinear gene pairs between *C. goeringii* and *D. catenatum*. The observed collinear relationship among the majority of the GRAS genes across the three orchid species suggests a conserved genomic arrangement within this gene family. Moreover, the correspondence observed between the GRAS genes of *C. goeringii* and those of the other orchids implies a shared evolutionary history or sequence similarity. Collectively, these findings indicate a substantial degree of homology among the GRAS genes in the three orchids. Additionally, analysis of synteny uncovered two pairs of segmental duplications. Further examination was conducted on these gene pairs to analyze selection pressure. The results suggest that all *CgGARS* genes experienced purifying selection (*Ka*/*Ks* < 1) (Table S3).

**Figure 5:**
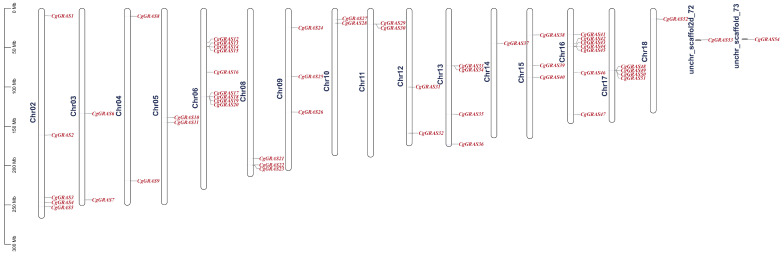
GRAS gene distribution on the chromosomes of *C. goeringii*.

Based on the annotated file of the C. goeringii genome, a visual analysis of the genes on chromosomes was performed (Figure 5). The results revealed that 54 genes were spread across 16 chromosomes, with the remaining two genes located on unidentified chromosomes. Chr06 has the most GRAS genes, with nine genes, Chr14 and Chr18 each contain only one gene, Chr03, Chr04, Chr05, Chr10, Chr11 and Chr12 each contain only two genes. The positioning of GRAS genes on the chromosomes of C. goeringii is predominantly clustered in the top and middle parts. Interestingly, there were tandem repeats on Chr06, Chr11, Chr13, Chr16, and Chr17.

### 2.5. Cis-Acting Regulatory Elements and Gene Ontology Analysis of GRAS Genes

**Figure 6:**
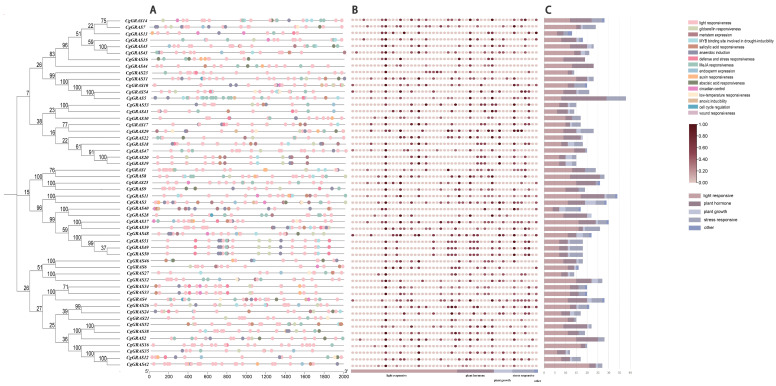
Promoter analysis of the *CgGRAS* genes. (**A**) The distribution of *cis*-acting elements at 2000 bp upstream of the GRASs. (**B**) The number of *cis*-acting elements in the promoter region. (**C**) The number of light response, plant hormone, plant growth, and stress response elements for each GRAS gene. The captions are marked on the right, and the types and quantities of cis-acting elements are shown in Table S5.

To identify potential cis-acting elements and further investigate the regulatory functions of GRAS genes, we searched cis-elements in the 2000 bp promoter region upstream of 54 genes in C. goeringii (Figure 6A). This search yielded 1128 cis-acting regulatory elements, encompassing light response, plant growth, plant hormone, and stress response categories (Table S4). Among them, Box4 elements related to light response had the largest number with 175 cis-acting regulatory elements, followed by CGTCA-motif and TGACG-motif elements related to plant hormone with 82 elements each (Figure 6B). Five types of elements correlated with plant growth were identified, totaling 57 (5.05%); ten types of plant hormone-related elements amounted to 309 (27.39%); 29 light response-related elements tallied 598 (53.01%); and six stress response-related elements accounted for 117 (10.37%). CgGRAS5 exhibited the highest element count at 38, while CgGRAS35 had the fewest at 12. CgGRAS5 harbored the most stress response-related cis-elements. Among the total, 54 genes contained cis-acting elements linked to light response and plant hormones. Conversely, nine genes, such as CgGRAS8, CgGRAS36, and CgGRAS52, lacked stress response-related cis-acting elements.

**Figure 7:**
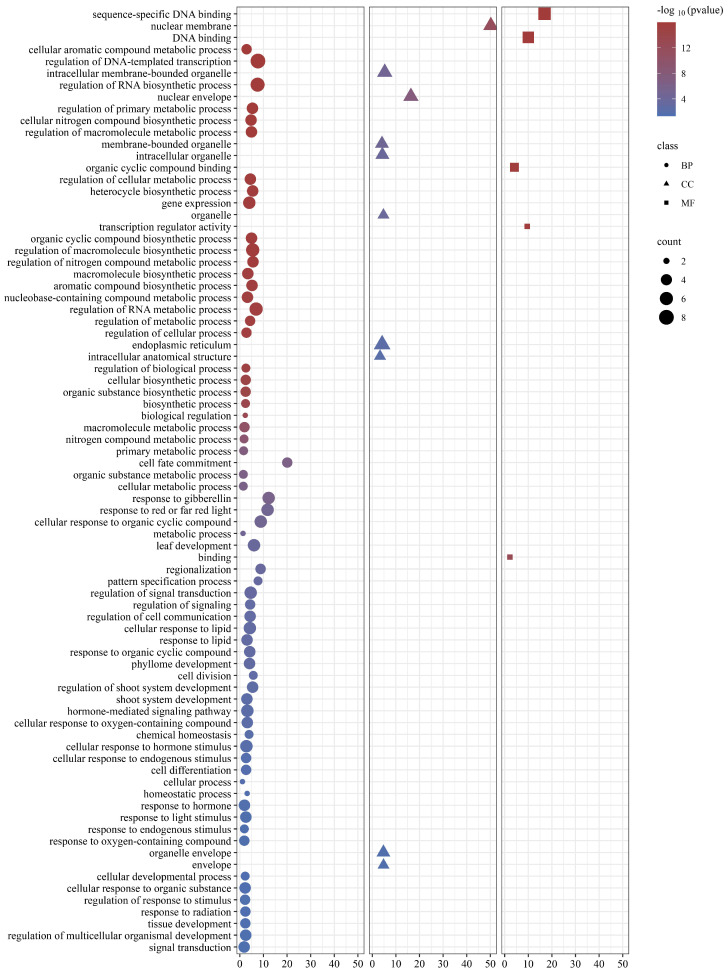
Gene ontology (GO) terms of CgGRAS. BP (Biological Processes), CC (Cellular Constituents), and MF (Molecular Functionalities).

To unravel the functional intricacies of GRAS proteins across diverse biological phenomena within *C. goeringii*, a meticulous Gene Ontology (GO) annotation analysis was conducted on the *CgGRAS* gene (Figure 7). The findings indicated that GRAS proteins could potentially participate in numerous biological processes, cellular constituents, and molecular functionalities. The identified associations were found to exhibit diverse functionalities, encompassing metabolic process regulation, biosynthesis, response to abiotic stimuli, and hormones.

### 2.6. The qRT-PCR Analysis of CgGRASs

**Figure 8:**
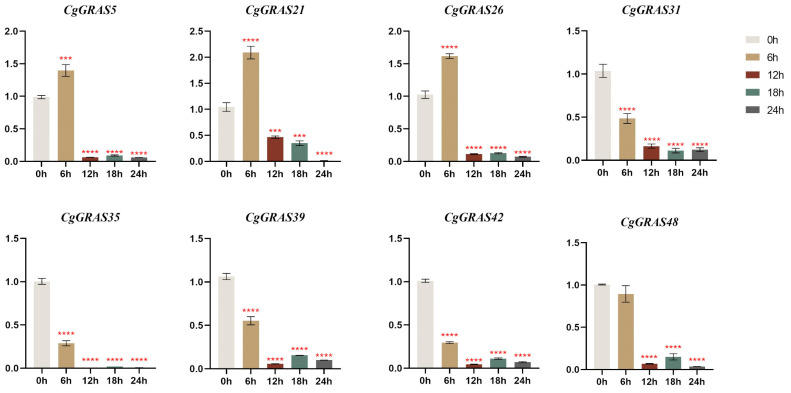
Real-time reverse transcription quantitative PCR (RT-qPCR) validation of 8 CgGRAS genes under high temperature stress. 0 h: control sample before high-temperature stress; 6 h: after 6 h of high-temperature stress; 12 h: after 12 h of high-temperature stress; 18 h: after 18 h of high-temperature stress; 24 h: after 24 h of high-temperature stress. The Y-axis represents relative expression values. Bars represent the mean values of three technical replicates±SE. Red asterisks indicate significant upregulation of corresponding genes after high-temperature stress treatment (*** *p* < 0.001, **** *p* < 0.0001, Student’s test). Primers are shown in Table S6. The FPKM value of *CgGRAS*s are shown in Table S7.

To examine the expression patterns of the *CgGRAS* gene under heat stress, we conducted RT-qPCR analyses on eight genes selected from various subfamilies. In the present study, qRT-PCR experiments were performed to analyze the expression patterns of the 8 *CgGRAS* members in leaves in response to heat treatment (Figure 8). Of particular note is the rapid increase in expression levels observed for three *CgGRAS* genes (*CgGRAS5*, *CgGRAS21*, and *CgGRAS26*) at 6 h post heat stress, with *CgGRAS21* showing particularly notable upregulation. Whereas the expression of all eight genes was significantly reduced during the 12, 18, and 24 h after the heat stress. Following 12 h of treatment, the expression levels of five genes (*CgGRAS31*, *CgGRAS35*, *CgGRAS39*, *CgGRAS42*, and *CgGRAS48*) were diminished in comparison to those observed after 6 h of treatment. However, subsequent to 18 h of treatment, a slight increase in the expression of these genes was observed except for *CgGRAS31*. The lowest expression levels of DELLA subfamily members *CgGRAS35* and *CgGRAS42*, along with the PAT1 subfamily member *CgGRAS39*, were observed at 12 h. In contrast, *CgGRAS48* from the PAT1 subfamily exhibited its minimum expression level after 24 h of-high temperature treatment. The expression of *CgGRAS31* (HAM subfamily) remained largely unchanged after 12 h but declined to its minimum at 18 h.

## 3. Discussion

Orchidaceae stands as one of the most expansive and species-diverse families among the flowering plants, and as plant-specific transcription factors, GRAS proteins are essential in a multitude of developmental processes for tissues and organs [2,31]. Over the past few years, considerable attention has been directed towards investigating the molecular mechanisms governing growth and development in *C. goeringii*, as well as the intricate processes involved in the regulation of its flower development [32,33]. However, the investigation into the GRAS gene family within orchids remains limited. This investigation delved into the evolution and functional characteristics of *CgGRAS* proteins employing a wide range of methodologies, thereby delineating the GRAS gene ensemble in *C. goeringii*.

In this study, we uncovered 54 *CgGRAS* genes within the genome of *C. goeringii*, a number similar to that reported in the genomes of tomato (53) [34], grapevine (52) [35], tobacco (53) [36], and strawberry (54) [37]; more than *D. catenatum* (47) [5], *A. thaliana* (32) [7], and *P. mume* (45) [10], and less than cassava (77) [38], soybean (117) [9], and *Malus domestica* (127) [39]. The *A. thaliana* genome contains 34 GRAS members, while the *C. goeringii* genome possesses 54 *CgGRAS* genes, suggesting that there has been an evolutionary expansion of the GRAS gene family in *C. goeringii*. Such variation observed in the numbers of GRAS genes could stem from differing gene duplication events or the impact of the overall genome size in these varied species [35].

As a result of our research, we identified 54 GRAS genes within the genome of *C. goeringii*, and these genes have been systematically classified into ten subfamilies (Figure 1). In our study, the HAM subfamily emerged as the most populous, comprising 13 out of the 54 GRAS genes identified. HAM protein has been implicated in the essential function of maintaining both apical and lateral meristems within *Petunia hybrida* [2]. Additionally, this subfamily’s members play an essential role in maintaining shoot meristem upkeep through their mediation of signals from differentiating cells [40]. Elevated expression of a PAT1 subfamily member derived from wild grape significantly boosts abiotic stress resistance in *A. thaliana* [41]. The GRAS members grouped within the same subfamily or clade may suggest their similar functions across different species. PAT1 subfamily members may have a vital role in enabling *C. goeringii* to withstand abiotic stresses like cold and photooxidative stress. In the vascular tissues of *A. thaliana*, SHR and SCR subfamily proteins jointly contribute to the specification of endodermis, and these proteins are inclined to constitute the SCR/SHR complex, which influences root radial organization [14,42,43]. It is known that DELLA proteins can stimulate gibberellic acid (GA) signal transduction and may influence the regulation of other phytohormones [44,45]. In this study, the LAS (2/54), LISCL (4/54), SCL3 (3/54), and SCL4/7 (1/54) subfamilies also had several members. These subfamilies are known to play pivotal roles in other plants in aiding in the management of abiotic stresses, responding to a variety of signals, and contributing to plant development [4]. Some specific subfamilies of GRAS proteins have diverged due to contraction or expansion processes during their evolutionary development. In the case of the Os19 subfamily (2/54), which is exclusive to monocot plants, the phylogenetic analysis conducted in this study reveals that it consists of merely two members, namely *CgGRAS6* and *CgGRAS46* [12].

The distinct domains, or conserved motifs, within GRAS proteins were crucial for maintaining their characteristics in protein interactions and DNA-binding modifications [46]. GRAS gene products are distinguished by their variable N-terminal region and a remarkably conserved C-terminal region. Furthermore, the complete preservation of the residues within the VHIID and SAW domains implies their indispensability for the functionality of GRAS gene products [47]. In the C-terminal region of GRAS proteins, the VHIID domain is notably the most conserved. Sequence alignment of the *CgGRAS* gene from this research reveals that almost all members contain the VHIID domain (Figure 3). Significantly, it can be observed that a range of amino acid substitutions is evident in almost every subfamily. For instance, every member of the DELLA subfamily possessing a complete motif backbone exhibits a substitution of isoleucine (I) with valine (V), whereas every member of the LAS subfamily shows a replacement of valine (V) with isoleucine (I) and isoleucine (I) with leucine (L). Additionally, the DELLA and LAS subfamilies are instrumental in fostering meristem development, overseeing GA signal transduction, and bolstering defense against stress [48,49]. The SAW motif is sequentially arranged into three units: WX7G, LW, and SAW, with the majority of *CgGRAS* genes in this study featuring this motif [50]. Despite the functions of SAW motifs being mostly uncharted, the consistent presence of certain residues in the SAW motifs implies a probable necessity for these motifs in uploading the structural coherence of GRAS proteins [4].

The earliest eukaryotic ancestors possessed a wealth of introns, which have been progressively lost over time, suggesting the significant contribution of introns to the plant’s evolution [51,52]. Interestingly, most genes in *CgGRAS* families have no introns; all genes in the DELLA subfamily are intronless, particularly (Fiure 2C). Despite offering no perks in terms of recombination or aiding in species evolution, genes that do not contain introns frequently display a quick response to stress. And the vast majority of orchids previously reported are known to possess long introns; however, this research presents a case with the observation of merely three long introns. Earlier studies have demonstrated that the ancestors of each eukaryote possessed genes rich in introns. It suggested that the significant loss and insertion of introns in most genes could be attributed to selective pressure, potentially expedited by gene duplication [53,54].

Throughout evolutionary history, plant genomes have witnessed the manifestation of ancient duplication events, engendering a significant surplus of duplicate genes. The presence of duplicate genes has played a pivotal role in evolutionary processes, facilitating the emergence of novel functions, including adaptation to varying environmental stressors [55]. Through collinear analysis, this study unveils the evolutionary connections among the GRAS genes in three orchid species (Figure 4). The observation of an almost one-to-one correspondence in the chromosomes of *D. chrysotoxum* and *C. goeringii* suggests minimal structural change between the chromosomes of these two species. Additionally, our analysis of *C. goeringii* GRAS gene duplications revealed 21 tandem duplication genes and only two pairs of segmental duplications. This prevalence of tandem duplications suggests that the evolution of *CgGRAS* genes is primarily driven by tandem duplication events.

*Cis*-acting elements, located within gene promoter regions, play a pivotal role in managing transcription and gene expression, as well as in responding to abiotic stress [56,57]. In this current investigation, we undertook a comprehensive analysis of the cis-acting regulatory elements within the promoter regions of the 54 *CgGRAS* genes identified. This analysis led to the discovery of some *cis*-acting elements that are linked to responses to stress. As is shown in Figure 6, *cis*-regulatory elements that are associated with stress, light response, plant hormones, and plant growth broadly exist. Numerous *cis*-regulatory elements associated with the regulation of GA signaling and hormone metabolism under biotic and abiotic stress conditions have been identified in other plants that have been studied [30,58], which coincides with the identification of our study. Furthermore, a group of the GRAS proteins shows significant enrichment in the regulation of response to stimulus based on the GO analysis (Figure 7). These findings of this research propose that *CgGRAS* genes likely assume a multifaceted function in *C. goeringii*, including involvement in the plant’s reaction to various stress factors and the regulation of diverse biological processes. This indicates the broad functional significance of these genes within the organism’s physiological and developmental contexts.

Plant-specific transcription factors within the GRAS family are pivotal for plant development [1]. The response mechanism under drought, salt, and low temperature stress has been extensively studied, but its specific functions and regulatory mechanisms under heat stress remain understudied, particularly in orchids. This study examined the response of eight *CgGRAS* genes from different subfamilies in leaves to heat stress (Figure 8). Following 4 h of elevated temperature exposure, the expression of the *BoGRAS* gene was notably enhanced in Brassica oleracea [59]. The expression of *CgGRAS5*, *CgGRAS21*, and *CgGRAS26* showed a significant increase after 6 h of heat stress, followed by a subsequent decrease. Conversely, members of the DELLA subfamily (*CgGRAS35*, *CgGRAS42*), the PAT1 subfamily (*CgGRAS39*, *CgGRAS48*), and *CgGRAS31* of the HAM subfamily exhibited a consistent downward expression trend. The ortholog of *CgGRAS5* in grapevine, *VviHAM3*, exhibits upregulation in response to drought stress in seeds and stems [35]. This expression pattern suggests that members of the HAM subfamily are involved in a broad range of stress responses. The LAS gene primarily functions in plant meristematic tissues, lateral organs, and leaf morphology. Moreover, overexpression of BnLASs in *A. thaliana* enhances its drought resistance. This study observed a significant upregulation in the expression level of *CgGRAS26*, belonging to the LAS subfamily, was significantly upregulated at 6 h under heat stress, indicating the potential involvement of the LAS subfamily in response to heat stress and enriching the exploration of the biological functions of the members of the LAS subfamily. SCL4/7 subfamily members are usually involved in inflorescence stage development, and *CgGRAS21* (SCL4/7 subfamily) showed significant up-regulation under high-temperature stress in our study. It suggested that it plays a role in resistance to high-temperature stress, providing a new direction for the exploration of the functions of SCL4/7 subfamily members. The orthologous gene of *HvGRAS30*, *CgGRAS21*, similarly exhibits a significant upregulation. The majority of GRAS genes in castor beans are suppressed after high-temperature stress [60], and in this study, the genes were also found to be downregulated after 12 h of heat stress, showing widespread inhibition of expression. *CgGRAS5* of the HAM subfamily was significantly upregulated after 6 h of heat stress. We found that after heat stress, most of these members exhibited significant differential expression, indicating coordinated responses and important roles in tolerance. This provides new clues for further exploration of the regulatory mechanisms governing the response of orchids to heat stress.

## 4. Materials and Methods

### 4.1. Data Sources

We acquired the genome sequence of *C. goeringii* and the annotation files from the National Center for Biotechnology Information (NCBI) under accession number PRJNA664445. Additionally, we downloaded the protein sequence of GRAS of *A. thaliana* and *O. sativa* from Tair and Phytozome v13, respectively.

### 4.2. Treatment of Plant Materials

The plant materials utilized in this study were obtained from the Forest Orchid Garden of Fujian Agriculture and Forestry University. Three pots of mature *C. goeringii*, all with matching growth periods and cultivation conditions, were chosen in their natural growth state and subjected to heat stress treatments within an artificial climate culture room. The plants were exposed to a photoperiod of 16 h light/8 h dark, 30 °C/38 °C; samples were taken at 0, 6, 12, 18, 24 h, respectively.

### 4.3. Identification and Physicochemical Properties of the GRAS Proteins

A local BLASTp search was conducted using *A. thaliana* GRAS proteins as a seed file (built-in TBtools) [61]. And the conserved domain PF03514 of GRAS was obtained from the online database (http://pfam.xfam.org/, accessed on 5 February 2024) for an HMMER search (default parameters) to screen for candidate GRAS gene family members in *C. goeringii* [62]. The results from BLAST and HMMER were intergrated to eliminate incomplete and redundant protein sequences. Furthermore, uncertain genes underwent a BLASTp search on the NCBI website. Protein analysis, including protein length, isoelectric point (pI), molecular weight (MW), hydrophilic large average (GRAVY), instability index (II), and fat index (AI), was performed using the online software ExPASy (https://web.expasy.org/protparam/, accessed on 5 February 2024) [63].

### 4.4. Phylogenetic Analysis of GRAS Genes

The protein sequences of GRAS from A. thaliana (34 *AtGRAS*s), O. sativa (60 *OsGRAS*s), and *C. goeringii* (54 *CgGRAS*s) were imported into the MEGA 7.0 software. For multi-sequence alignment, we utilized the muscle program with default settings, and the neighbor-joining (NJ) method was employed to construct the phylogenetic tree comprising 148 protein sequences (bootstrap method: 1000). Furthermore, we utilized the online software Evolview3.0 (https://evolgenius.info//evolview-v2/, accessed on 5 February 2024) to enhance and refine the phylogenetic tree [64].

### 4.5. Gene Structure and Motif Analysis of CgGRASs

We analyzed the conserved domains of GRAS genes using the CDD tool available in the NCBI online software (https://www.ncbi.nlm.nih.gov/Structure/bwrpsb/bwrpsb.cgi, accessed on 5 February 2024). Additionally, we employed the online software MEME (http://meme-suite.org/, accessed on 17 February 2024) to analyze and download the conserved motifs of the GRAS gene in *C. goeringii*, with the prediction number set to ten [65]. For integrating phylogenetic trees, conserved protein motifs, and overall comparative maps of gene structure, we utilized TBtools v1.120.

### 4.6. Collinearity and Chromosomal Localization of CgGRASs

We utilized the One-Step MCScanX utility from TBtools v1.120 to examine the collinear relationships among *C. goeringii*, *D. chrysotoxum*, and *D. nobile* and to pinpoint the collinear gene blocks of GRAS within their genomic sequences.

To analyze the chromosomal localization of GRAS genes in *C. goeringii*, we utilized the TBtools v1.120 (Chengjie Chen, Guangzhou, Guangdong 510640, China) software to extract location information from the genome and gene annotation files. Subsequently, we constructed the physical map of GRAS genes on the chromosome [61].

### 4.7. Cis-elements and Gene Ontology Analysis of CgGRASs

To identify putative *cis*-acting elements in the promoter, the 2000 bp regions upstream of the GRAS genes in *C. goeringii* were extracted by TBtools [66]. The online software PlantCARE (https://bioinformatics.psb.ugent.be/webtools/plantcare/html/, accessed on 5 February 2024) [67] was used to analyze the *cis*-acting regulatory elements in the promoter region of the *CgGRAS* gene. Subsequently, we processed the data using Excel 2018 software (Charles Simonyi, Redmond, State of Washington, the United States) and visualized it using TBtools v1.120 and Origin (https://www.originlab.com/Origin, accessed on 5 February 2024) online software.

The gene ontology (GO) is a globally standardized system for classifying gene functions, employed in conducting functional enrichment analysis of differentially expressed genes to identify enriched functionalities among them. Employing the *C. goeringii* genome GO protein files, retrieval was conducted within the Uniprot (Universal Protein) database. Utilizing the GO Seq R package, GO enrichment analysis was performed on the GRAS gene family members within *C. goeringii*, unveiling their potential involvement across a spectrum of biological processes, cellular components, and molecular functions.

### 4.8. RT-qPCR Analysis

We extracted total RNA from the leaves of *C. goeringii* using the RNA Simple Plant Kit. Subsequently, we synthesized first-strand DNA with TransScript^®^ All-in-One First-Strand cDNA Synthesis SuperMix for quantitative PCR (qPCR; TransGen Biotech, Beijing, China). To ensure the removal of genomic DNA, the same TransScript^®^ All-in-One First-Strand cDNA Synthesis SuperMix (TransGen Biotech, Beijing, China) was utilized for qPCR. Primers for RT-qPCR targeting *CgGRAS* were designed using Primer Premier 5 software, and their specificity was confirmed through primer blast on the NCBI website. RT-qPCR analysis was conducted using PerfectStart™ Green qPCR SuperMax (TransGen Biotech, Beijing, China). In this study, the *Actin* gene from *C. goeringii* served as the reference gene. The relative expression of the target gene was determined using the 2^−ΔΔCT^ method. All the RT-qPCR analyses were performed with three technical replicates each.

## 5. Conclusions

Our study reported the GRAS gene family of C. goeringii for the first time. 54 CgGRASs were identified in the whole genome of C. goeringii, and their physicochemical attributes, phylogenetic associations, gene structure, cis-acting elements, and expression profiles under heat stress. The analysis provided a reference for further analysis of the heat stress function of the GRAS gene family in the future. This study analyzed the mechanism of the GRAS gene in response to biological stress, but GRAS protein and plant hormones play a key role in plant growth and stress signaling, so the interaction between GRAS and plant hormone induction genes needs to be further studied in the future.

## Data Availability

The genome sequence of *C. goeringii* and the annotation files were downloaded from the National Center for Biotechnology Information (NCBI) (accession number: PRJNA749652). And the protein sequence of GRAS of *A. thaliana* and *O. sativa* were downloaded from Tair (https://www.arabidopsis.org, accessed on 1 February 2024) and Phytozome v13 (https://phytozome-next.jgi.doe.gov, accessed on 1 February 2024), respectively.

## Supplementary Materials

The following supporting information can be downloaded at: https://www.mdpi.com/article/10.3390/ijms25126363/s1.
